# Predicting Abdominal Aortic Aneurysm Target Genes by Level-2 Protein-Protein Interaction

**DOI:** 10.1371/journal.pone.0140888

**Published:** 2015-10-23

**Authors:** Kexin Zhang, Tuoyi Li, Yi Fu, Qinghua Cui, Wei Kong

**Affiliations:** 1 Department of Physiology and Pathophysiology, School of Basic Medical Sciences, Peking University, Beijing, P. R. China; Key Laboratory of Molecular Cardiovascular Science, Ministry of Education, Beijing, P. R. China; 2 Department of Bioinformatics, School of Basic Medical Sciences, Peking University, Beijing, P. R. China; IIBB-CSIC-IDIBAPS, SPAIN

## Abstract

Abdominal aortic aneurysm (AAA) is frequently lethal and has no effective pharmaceutical treatment, posing a great threat to human health. Previous bioinformatics studies of the mechanisms underlying AAA relied largely on the detection of direct protein-protein interactions (level-1 PPI) between the products of reported AAA-related genes. Thus, some proteins not suspected to be directly linked to previously reported genes of pivotal importance to AAA might have been missed. In this study, we constructed an indirect protein-protein interaction (level-2 PPI) network based on common interacting proteins encoded by known AAA-related genes and successfully predicted previously unreported AAA-related genes using this network. We used four methods to test and verify the performance of this level-2 PPI network: cross validation, human AAA mRNA chip array comparison, literature mining, and verification in a mouse CaPO_4_ AAA model. We confirmed that the new level-2 PPI network is superior to the original level-1 PPI network and proved that the top 100 candidate genes predicted by the level-2 PPI network shared similar GO functions and KEGG pathways compared with positive genes.

## Introduction

Abdominal aortic aneurysm (AAA) is characterized by permanent abdominal aortic dilation, with the maximum diameter of diseased aorta reaching 1.5 times that of the adjacent aorta. Due to a lack of apparent signs and symptoms, most AAAs, when first diagnosed, are at risk of rupture and hemorrhage with very high fatality rate. This complex cardiovascular disease is modulated by multiple genes related to extracellular matrix degradation, oxidative stress, inflammation, and apoptosis [1]. To date, the only clinical treatment of AAA is invasive surgical repair, and no proven drug therapy is available. Exploring novel anti-aneurismal candidate genes could shed light on promising strategies for AAA prevention and therapy.

In general, functionally related genes are likely to cluster in the same networks [2]. Analysis revealed that genes related to a particular disease tend to have higher and more synchronized expression and tend to interact among each other [4] [5, 6]; approximately 70–80% of proteins share at least one function with an interacting partner [7]. Furthermore, proteins of similar function and cellular location tend to cluster together. Approximately 63% of the interactions occur between proteins with common functions, and 76% occur between proteins in the same subcellular compartment [8].

Most protein-protein interaction (PPI)-based bioinformatics studies for predicting disease related genes are based on direct PPIs, although the systems and dimensions considered vary. A machine-learning approach [9] to analyzing protein-protein interaction data has become popular and has been applied to diverse biological problems, including gene classification [10], prediction of function, and cancer tissue classification. Some approaches used to predict disease genes are based on using combined PPI network topological features [11, 12] to construct a combined classifier, or on analysis of protein sequences [5]. The candidate genes predicted by these methods must have at least one direct interaction with a known disease gene. Thus, the scope of annotation for a candidate gene is limited by the annotation of its interacting partners [13]. Therefore, previous research using level-1 PPI networks to predict candidate disease genes may omit genes without direct interactions with known disease genes but that share substantial functional similarities with level-2 neighbors [13].

Recently, it was shown that if two proteins do not interact directly but share more common interacting partners than two proteins chosen at random, these two proteins are likely to have close functional associations [14]. This is referred to as indirect functional association. Through the use of indirect interaction data and topological weight, researchers are able to augment the protein-protein interaction network, thereby improving the precision of clusters predicted by existing clustering algorithms [15]. Chua and colleagues reported the use of indirect protein-protein interactions between level-2 neighbors for the prediction of protein complexes [15]. However, whether this method can be extended to the prediction of human disease gene candidates remains elusive.

We propose a novel method to predict candidate AAA disease genes by assessing indirect protein interactions. By comparison with the original PPI network, we found that using a novel level-2 PPI network is superior to the original method in terms of both quantity and quality of the predicted candidate genes. Moreover, we verified our results *in vivo* in a CaPO_4_-induced aneurysm mouse model.

## System and Methods

### 2.1 Data Sources and Preprocessing

**Fig 1:**
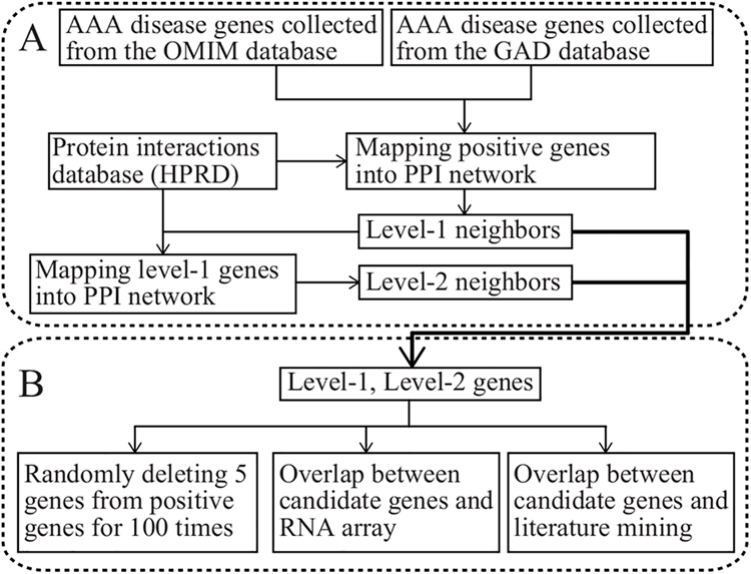
An illustration of the workflow: construction of the PPI network (A) and validation using various methods (B).

Genes defined as Abdominal Aortic Aneurysm (AAA) disease genes were compiled from the Online Mendelian Inheritance in Man database [16] (OMIM, http://www.ncbi.nlm.nih.gov/omim/) and Genetic Association Database [17] (GAD, http://geneticassociationdb.nih.gov/). PPI data from the Human Protein Reference Database [18] (HPRD, http://www.hprd.org/) were manually extracted from the literature by expert biologists who read, interpreted and analyzed the published data. To avoid any bias toward well studied genes [19], we examined the PPI networks for all interactions detailed by the HPRD annotation (Fig 1).

### 2.2 Candidate Gene Score Counting Method

The interaction number between each candidate gene (both level-1 and level-2) and a positive gene is designated as follows: node “A” indicates a positive gene, while nodes “B,” “C,” and “D” indicate level-1 candidate genes and nodes “E” and “F” indicate level-2 candidate genes. The interaction number between node B, C or D and positive gene node A is 1; the interaction number between node E and positive gene node A is 1; and the interaction number between node F and positive gene node A is 3 (S1 Fig).

### 2.3 Prediction of Candidate Genes

We constructed both level-1 and level-2 PPI networks to predict AAA disease genes (candidate genes). In the level-1 PPI network, the direct neighbors of all positive disease genes were named level-1 candidate genes. In the level-2 PPI network, the indirect neighbors (proteins with common interaction partners) of all positive disease genes were named level-2 candidate genes.

### 2.4 Animal Preparation

The animal experimental protocol was approved by the Institutional Animal Care and Use Committee (IACUC) of the Peking University Health Science Center (LA2015–142). All studies followed the guidelines of the Animal Care and Use Committee of Peking University. Appropriate analgesics were used on all animals to reduce surgical pain. Mice undergoing surgery to induce abdominal aortic aneurysm were anesthetized by intraperitoneal injection of pentobarbital (30–40 mg/kg) according to IACUC recommendations. After surgery, the animals were moved to a dry area with warming pad and were monitored during recovery.

Mice AAA was induced by CaPO_4_ as previously described [20]. For the experimental group (n = 7), the infrarenal aortas of C57BL/6J mice (male, 12 weeks, purchased from Vital River, Beijing, China) were isolated and wrapped with gauze presoaked in 0.5 mol/L CaCl_2_ for 10 minutes. Then, the CaCl_2_-treated gauze was substituted with gauze presoaked in PBS for another 5 minutes. The abdominal cavities were washed with 0.9% NaCl before suturing. For the sham operation group (n = 7), both CaCl_2_ and PBS solution were substituted with 0.9% NaCl solution, and all the other operations were same as the experimental group. Aortas were collected 7 days later. A picture of the aortas together with a dividing ruler was taken, and the maximum diameter of the aortas was measured using Image Pro Plus 6.0 according to the ruler.

### 2.5 Morphology of Aortas

Mice were killed, infrarenal aortas were perfused with PBS and 4% paraformaldehyde. Serial cryosections (7-μm thick, 300 μm apart) were analyzed by Gomori staining for elastin assessment.

### 2.6 Immunofluorescence Staining

Frozen aortic sections were incubated with antibodies against IL-6 (1:200, Abcam) and MCP-1 (1:50, Abcam), followed by secondary FITC-conjugated goat anti-mouse IgG (1:300) (Rockland Inc., Gilbertsville, Pennsylvania). Nuclei were counterstained with Hoechst 33342. The negative control was the primary antibody replaced with IgG. The fluorescence signal was monitored by confocal laser scanning microscopy (Leica, Germany).

### 2.7 Real-Time PCR

Real-time PCR amplification involved the use of an Mx3000 Multiplex Quantitative PCR System (Stratagene Corp, La Jolla, California) and SYBR Green I reagent. Products were normalized to an internal β-actin control. The primer sequences used for real-time PCR are provided in S1 Table.

### 2.8 Analysis of Functional Coherence

Cytoscape software [21] was used to visualize complex networks and to integrate attribute data. BINGO [22] was used to evaluate which Gene Ontology (GO, http://www.geneontology.org/) terms were enriched. We also tested whether candidate genes shared functions with positive disease genes to validate the associations between Top 100 level-2 candidate genes and the incidence of disease.

Web-based Gene Set Analysis Toolkit [2] (WebGestalt, http://bioinfo.vanderbilt.edu/webgestalt/) is an extensively used tool for functional enrichment analysis. We used it to compare level-2 candidate genes and positive disease genes with genes in KEGG pathways to identify significant pathways in which level-2 candidate genes and positive disease genes took part. A significance level of 0.01 was selected as the cutoff for selecting enriched pathway categories [11].

### 2.9 Statistical Analysis

All data are presented as the mean±standard error of the mean (SEM). Statistical analysis was performed with Student’s t-test for the CaPO_4_ AAA model. Cross validation was tested using the paired samples t-test. P<0.05 was considered statistically significant.

## Results

In this article, we established a novel method for generating level-2 PPI networks to identify candidate disease genes by measuring the number of shared interaction partners of each gene in a PPI network. This method is detailed in Fig 1.

### 3.1 Level-1 and Level-2 PPI Network Construction

AAA-related genes were compiled from the OMIM and GAD databases. Together, 56 positive genes were identified (33 genes from OMIM and 37 genes from GAD; 14 genes were found in both databases). These genes were entered into the HPRD database, and level-1 neighbors known to directly interact with the genes were identified. These level-1 neighbors were in turn entered into the HPRD database, and their level-1 neighbors—that is, the level-2 neighbors of disease genes—were found (Fig 1). In the HPRD database, 959 level-1 neighbors with 1,312 direct interactions and 5,730 level-2 neighbors with 42,615 indirect interactions were identified (Table 1). Level-1 neighbors, together with the disease genes and direct interactions, comprise the level-1 PPI network; level-2 neighbors, together with the disease genes and indirect interactions, comprise the level-2 PPI network. Of the 56 positive genes in the level-1 PPI network, only 34 had a direct interaction with another positive gene, accounting for 60.71% of all positive genes. Of the 56 positive genes in the level-2 PPI network, 52 had an indirect interaction with another positive gene, accounting for 92.86% of all positive genes.

**Table 1:** Main Features of Level-1 and Level-2 PPI Network.

	Level-1	Level-2
**Positive Gene Interactions**	34	52
**Positive Gene Interaction Percentage** (Positive Genes with Interactions/ Total Positive Genes)	60.71%	92.86%
**Candidate Genes**	959	5730
**Protein-Protein Interactions**	1312	42615

### 3.2 AAA-Related Gene Prediction and Topological Features of the PPI Network

**Fig 2:**
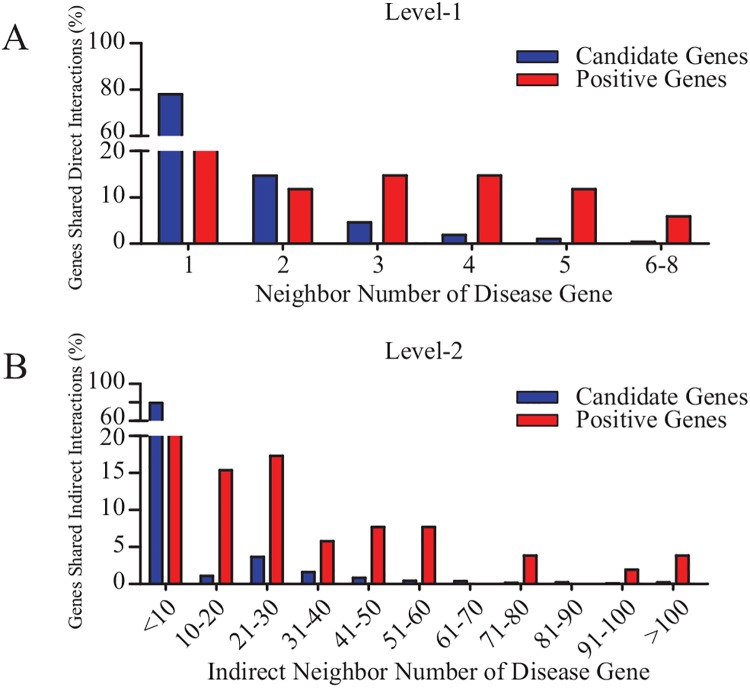
Probability distributions of level-1 and level-2 neighbors for positive and candidate genes.

Genes that encode proteins that interact directly or indirectly with the proteins encoded by known AAA-related genes were referred to as candidate genes. The number of interactions between every candidate gene and all known AAA related genes was calculated, and the interaction score of every candidate gene was then obtained. Based on the probability distribution, we found that in both level-1 and level-2 PPI networks, positive genes possessed much higher interaction scores with known AAA-related genes than did candidate genes (Fig 2). More precisely, most candidate genes had more than 3 direct interactions or less than 10 indirect interactions with known AAA-related genes; positive genes and candidate genes showed significant differences in distribution when the direct interaction number was greater than 4, or when the indirect interaction number was greater than 10. Thus, level-1 candidate genes with greater than 4 interactions are very likely to be hub nodes. The level-2 candidate genes and AAA disease genes may share physical or biochemical characteristics that allow them to bind to the hub nodes [3]. The more interaction partners they share, the higher the probability that they share a common function. In the level-1 PPI network, 14.7% of the positive genes shared at least 4 direct interactions with an AAA-related gene, an 8-fold increase compared with the candidate genes (1.9%) (Fig 2A). In the level-2 PPI network, 17.3% of the positive genes shared at least 50 indirect interactions with an AAA-related gene, an 11-fold increase compared with the candidate genes (1.6%). As seen in Fig 2B, most positive genes shared over 50 indirect interactions with AAA disease genes. Therefore, level-2 candidate genes with more than 50 indirect interactions were defined as prior candidate genes.

**Fig 3:**
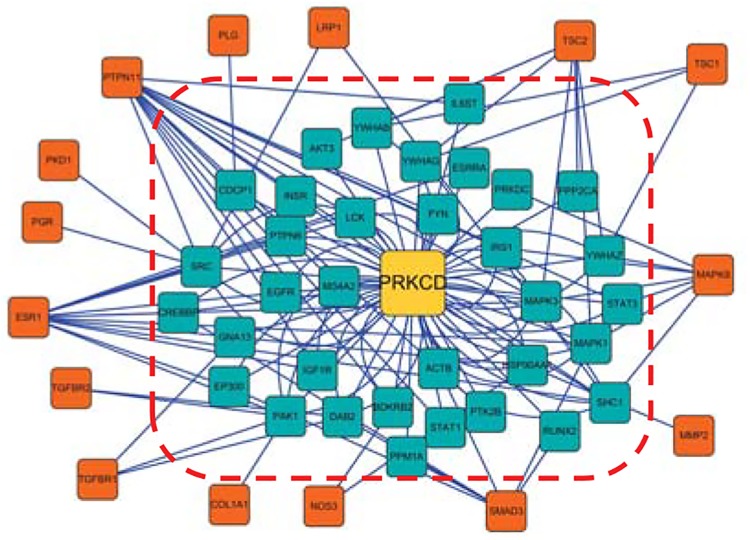
An example of indirect interaction with AAA disease genes. The PRKCD gene (labeled in yellow) is present as a root of trees in which the level-1 (labeled in blue) and level-2 neighbors (labeled in red) correspond to the level-1 and level-2 child nodes.

One of the prior candidate genes with over 50 indirect interactions and 0 direct interactions (i.e., not predicted by the level-1 PPI network) with AAA disease genes was chosen for further study. As shown in Fig 3, protein kinase C delta (PRKCD) was chosen as the center of a protein network. In this network, PRKCD had no direct link (i.e., interaction) with AAA disease genes (shown as red child nodes), but had common interacting partners (level-1 neighbors, shown as blue child nodes) with AAA-positive genes. Specifically, level-1 child nodes, such as the MAPK1, MAPK3, PAK1 and SHC1 genes, were likely to be hub nodes through which PRKCD and known AAA-related genes indirectly interact. In a published human AAA RNA chip array dataset (Reference Series: GSE7084, GSE47472) [23], PRKCD expression was significantly increased in AAA cases compared with controls. In addition, PRKCD expression was markedly upregulated in human AAA vessel wall and was shown to mediate VSMC MCP-1 expression [24], which could contribute to the vascular inflammatory process. Furthermore, recent studies in a mouse model of AAA have shown that PRKCD is an important signaling molecule in VSMC apoptosis and inflammation [25].

### 3.3 Cross-Validation

**Fig 4:**
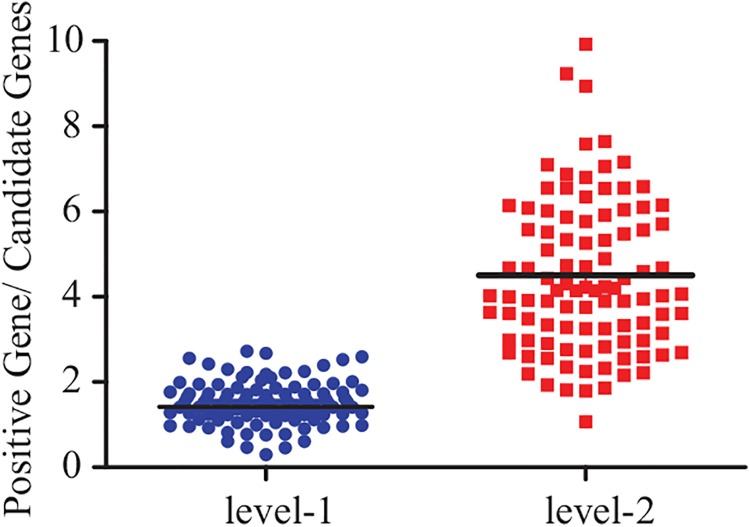
The ratio of positive genes to candidate genes. Five genes were randomly deleted from the set of 34 positive genes, and the PPI network was reconstructed 100 times.

A holdout validation test was used to evaluate the performance of both level-1 and level-2 PPI networks and to select the optimal PPI network. Five genes were randomly deleted from the list of 34 positive genes (i.e., positive genes shared by level-1 and level-2 networks), and both PPI networks were reconstructed. This process was repeated 100 times. The average scores for the positive and candidate gene groups were obtained each time and the ratios of the two average scores were calculated. In turn, the ratio after 100 randomizations was determined (Fig 4). According to this analysis, the ratio for the level-2 group was significantly higher than that for the level-1 group, making it more suitable for screening candidate genes related to AAA.

### 3.4 Comparison with Existing AAA Gene Data

**Fig 5:**
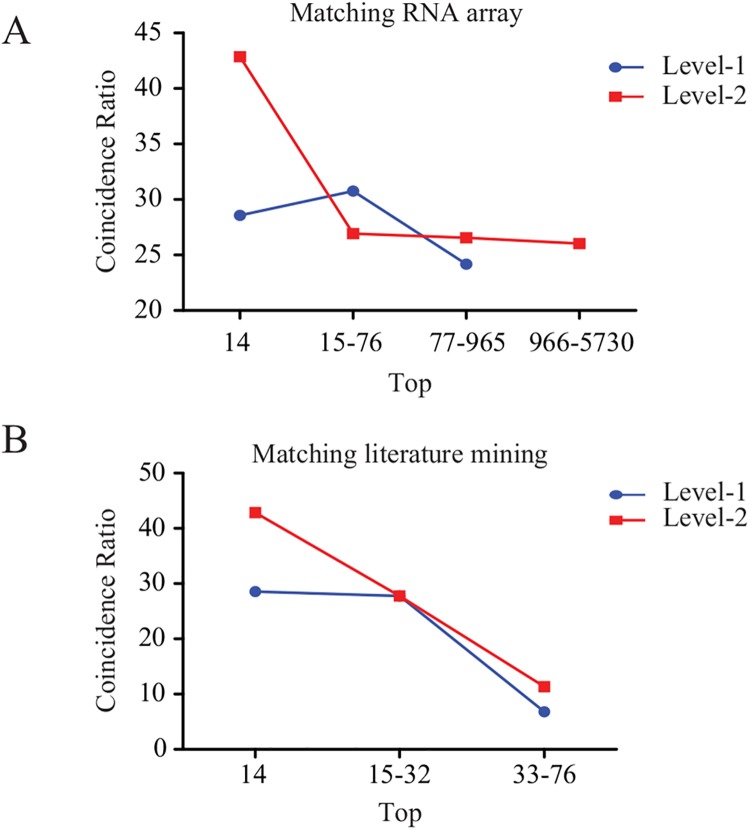
The coincidence ratios of the candidate genes to RNA array (A) and literature mining (B) for different top-level groups.

Human AAA RNA array data(Reference Series: GSE7084, GSE47472) obtained via Illumina and Affymetrix microarray platforms was used to generate global gene expression profiles for both aneurismal (AAA) and non-aneurismal abdominal aortas. The genes that were significantly differentially expressed between cases and controls were used in our analysis. Identified candidate genes from both level-1and level-2 PPI networks were matched with the RNA array data, and the coincidence ratios of the candidate genes to the RNA array data in different top-level groups were calculated. There were 3,274 differentially expressed genes, including 235 level-1 candidate genes (6.3%) and 1,240 level-2 candidate genes (33.1%). The top 14 genes corresponded to level-1 genes scoring greater than or equal to 5 and level-2 genes scoring greater than or equal to 100, with overlaps of 28.6% and 42.9% with the AAA chip array data, respectively. The top 15–76 genes corresponded to level-1 genes scoring 3–4 and level-2 genes scoring 82–98, with overlaps of 30.8% and 26.9% with the AAA chip array data, respectively. The top 77–965 genes corresponded to level-1 genes scoring 1–2 and level-2 genes scoring 12–31, with respective overlaps of 24.2% and 26.5% with the AAA chip array data. The remaining level-2 candidate genes scoring below 12 accounted for 26% of the genes. The upper range was segmented according to integer score. It was found that the coincidence ratio of level-2 candidate genes to the microarray data was significantly higher than that of level-1 candidate genes to the microarray data, especially among the top 14 (Fig 5A).

In addition, literature available in PubMed was reviewed to determine whether candidate genes predicted by the two PPI networks were related to AAA. The candidate genes in the level-1 group scoring greater than 4 and those in the level-2 group scoring greater than 50 were selected. According to the literature, predicted candidate genes in the level-2 group had a higher coincidence ratio with published studies though the top 10 to top 75 groups, as shown in Fig 5B. The top 14 genes corresponded to level-1 genes scoring greater than or equal to 5 and level-2 genes scoring greater than or equal to 100, with respective overlaps 28.6% and 42.9% compared to the AAA chip array data. The top 15–32 genes corresponded to level-1 genes scoring 4 and level-2 genes scoring 82–98, with respective overlaps of 28.6% and 42.9% compared to the AAA chip array data. The top 33–76 genes corresponded to level-1 genes scoring 3 and level-2 genes scoring 54–81, with respective overlaps of 28.6% and 42.9% compared to the AAA chip array data.

### 3.5 Analysis of Functional Coherence

Functional coherence between candidate genes and known disease genes was examined to verify associations between candidate genes and AAA. The KEGG database (WebGestalt, http://bioinfo.vanderbilt.edu/webgestalt/), which contains all signaling pathways in a Web-based Gene Set Analysis Toolkit platform, was utilized. The level-2 candidate genes and known AAA-related genes were entered, and a pathway enrichment analysis for both candidate genes and positive genes was performed. Eighty-five signaling pathways with enriched candidate AAA genes were obtained, with some—such as the ErbB signaling pathway, Focal Adhesion, Neurotrophin signaling pathway, TGF-β signaling pathway, MAPK signaling pathway, and Adherens Junction, B Cell Receptor signaling pathway (S3 Table)–showing marked enrichment.

Literature reports indicate that the TGF-β signaling pathway is involved in AAA. In animal models of AAA, TGF-β decreased aortic inflammatory cell infiltration, extracellular matrix degradation, and vascular smooth muscle cell apoptosis, thereby inhibiting AAA formation, progression and rupture. Active TGF-β binds to TGF-β Receptor II [25], which recruits and phosphorylates TGF-β Receptor I [26, 27]. The phosphorylated TGF-β Receptor I then activates receptor-regulated Smads [28], including Smad2 (a level-2 candidate gene) and Smad3 (a positive disease gene). In addition, inhibitory Smads (I-Smads) were identified [3]. One of these inhibitory Smads, Smad 7, negatively regulated TGF-β/ Smad signaling by preventing the activation of Smad2 and Smad3 [28, 29]. In our pathway enrichment analysis, Smad7 was identified as a level-2 candidate gene (S2 Fig).

### 3.6 Candidate Gene Expression in a CaPO_4_-Induced AAA Model

**Fig 6:**
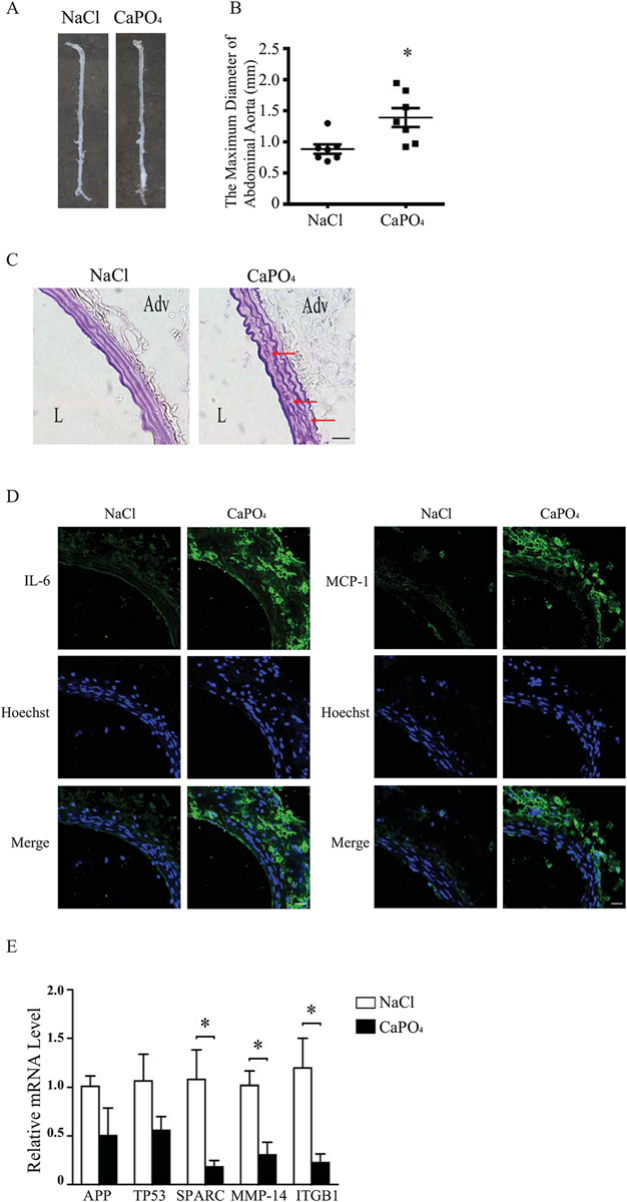
The mRNA expression levels of top-ranking candidate genes in a mouse AAA model. (A) Representative morphology of NaCl- and CaPO_4_-treated mice aortas. (B) The maximum diameter of abdominal aortas. (C) Representative Gomori staining, arrows showing elastin degradation. (D) Representative immunofluorescence (green) staining of IL-6 (left) and MCP-1 (right) in infrarenal aortas of mice treated with NaCl or CaPO_4_. Nuclei were counterstained with Hoechst (blue). (E) mRNA was extracted from the aortas of both NaCl- and CaPO_4_-treated mice, and the gene expression levels of APP, TP53, SPARC, MMP-14 and ITGB1 were assayed by Real-Time PCR. 18S was used as reference gene. Scale bar = 20 μm.

Five of the candidate genes at the top of the level-2 PPI network list that were unreported in the literature were tested and validated in a CaPO_4_-induced mouse abdominal aortic aneurysm model [30]. In the CaPO_4_-treated group, the infrarenal aorta was obviously expanded, while the aortas of NaCl-treated mice were morphologically normal (Fig 6A). The maximum diameter of the control (0.9% NaCl) group was 0.885± 0.077 mm, and that of the CaPO_4_-treated group was 1.391± 0.151 mm (p< 0.05) (Fig 6B). The elastic lamina disruption of the CaPO_4_-treated group was severer than that of the NaCl group as shown by Gomori staining (Fig 6C). Immunofluorescence staining showed that IL-6 and MCP-1 production in the aorta were also significantly increased in the CaPO_4_-treated group (Fig 6D). These data indicated the successful induction of infrarenal abdominal aortic aneurysm by CaPO_4_ treatment.

RNA was extracted from the abdominal aortic tissue of both groups and was reverse transcribed into cDNA. Five of the candidate genes predicted in the level-2 PPI network were verified using Real-Time PCR. Compared with the NaCl-treated group, CaPO_4_-treated aortas contained reduced levels of Osteonectin (SPARC), Matrix Metalloproteinase-14 (MMP-14) and Integrin beta 1 (ITGB1) mRNA expression, while Amyloid Precursor Protein (APP) and tumor protein p53 (TP53) showed no significant differences (Fig 6E).

## Discussion

Abdominal aortic aneurysm is a complex cardiovascular disease in which multiple genes are involved [31, 32]. In this study, we created a protein interaction database to predict AAA-related genes. We constructed a level-2 PPI network according to the interaction partners shared by positive genes and candidate genes, and successfully predicted a large number of undiscovered AA- related genes.

In recent years, the amount of available protein interaction data has markedly increased, and many protein interaction databases have emerged. Using various disease gene prediction methods, it has been found that even when fixed position candidate strategies or gene chip arrays are utilized, protein interaction networks yield additional candidate genes in a convenient and accurate manner [33]. Genes and their interaction partners are likely to share similar functions, and their reciprocal links are often closely related to certain phenotypes or diseases [34, 35]. Goh [3] and colleagues reported that proteins responsible for the same disease are inclined to cluster in a PPI network and that changes in neighbors of the disease genes are likely to contribute to the same or a similar disease. According to Josson [36] et al., the interaction probability between disease genes is much higher than that between non-disease genes, and disease genes are more prone to gather in clusters.

Direct protein-protein interaction (level-1 PPI) networks were widely used to predict the biological functions of proteins and disease related genes, especially in cancer and diabetes. However, at least one direct interaction with a known disease gene is required in a level-1 PPI network. Thus, genes that do not interact directly with known disease genes but share common interacting partners with those of a positive gene could be neglected.

In this study, we constructed a level-2 PPI network based on the number of shared interacting partners of known disease genes, then used it to predict candidate genes for abdominal aortic aneurysm (AAA). We found that most AAA candidate genes are likely to have the following characteristics: i) they tend to be hub nodes or to be bound to hub nodes in the network, indicating a greater link to disease genes; ii) they have a great number of indirect interactions with disease genes; iii) they have similar biological functions with known AAA genes according to GO functional categorization (S3 Fig); and iv) they often take part in common signaling pathways with known AAA related genes. According to our analysis, our network of 56 positive genes contains 959 level-1 neighbors and 5,730 level-2 neighbors, and previously identified candidate genes tended to be hub nodes. Our functional enrichment analysis and experimental validation confirmed this inference. In our CaPO_4_ induced mouse AAA model, 3 (SPARC, MMP-14 and ITGB1) of the 5 chosen prior candidate genes were significantly down-regulated in the AAA model compared with controls, and the other 2 genes (APP and TP53) tended to decrease.

Recently, some research on SPARC in intracranial aneurysm showed controversial results of SPARC expression in intracranial aneurysm [37, 38, 39]. However, we found for the first time that SPARC decreased in CaPO_4_ treated mice abdominal aorta.

For MMP-14, the reports are also contentious. Stéphanie Michineau *et*.*al*. conducted a CaCl_2_ model (14 days) to induce mice AAA, and reported that MMP-14 increased [40]. But in Hazem Abdul-Hussien's study, using tissue from patients, they found that a modest augment of MMP-14 was observed only in ruptured aneurysm. While in growing (unruptured) aneurysm, MMP-14 mRNA level was the same as that of normal controls [41]. In our study, the mice were sacrificed 7 days after CaPO_4_ treatment, and the aortas remained unruptured. We found that MMP-14 was down regulated in CaPO_4_ treated group 7 days after surgery. These discrepancies could probably due to experimental conditions like various animal model, CaCl_2_ incubation time, the induction of PBS, days after surgery, animal age etc.

Some previously reported genes that influence AAA, such as Epidermal Growth Factor Receptor (EGFR) [42], Smad family member 1 (Smad1) [43], Estrogen Receptor (ESR2) [44], and STAT family member 3 (STAT3) [45], were successfully predicted by our level-2 PPI network, but not by the level-1 PPI network. These reported genes were not positive in our network because they were identified in animals, whereas only human genome-wide association study (GWAS) reports were used in the construction of our networks.

Two difficulties remain in the prediction of disease genes based on PPI networks. One is the current inadequacy of PPI databases; approximately one-third are based on computer prediction, creating concerns regarding the reliability of the data. The other difficulty is that network topological features are difficult to quantitate. For example, a protein with a high degree might be a hub node, but it might also be a well-studied protein of lesser importance.

Further investigation of prior candidate genes and their interactions with positive genes may shed light on AAA nosogenesis and its underlying mechanisms. Future work with multiple AAA models and experimental methods is necessary to ascertain whether candidate genes are truly AAA-related and how they play a role in the disease.

## Conclusions

We constructed a level-2 PPI network based on indirect (level-2) PPI neighbors with common interaction partners of known disease genes. The more common interaction partners a gene possessed, the higher the chance that the two proteins would share certain common functions. Select level-2 candidate genes were screened, and those with more than 50 indirect interactions with positive disease genes were referred to as prior candidate genes. These prior candidate genes were found to share similar GO functions and KEGG pathways with positive disease genes. Thus, predicting disease-related genes using a level-2 PPI network based on indirect protein-protein interactions can serve as a guide for researchers to discover additional novel disease-related genes.

## Supporting Information

S1 Fig Candidate gene score counting method. (EPS)

S2 Fig The detailed interaction of candidate disease genes in the KEGG pathway and the TGF-beta signaling pathway. Blue nodes represent candidate genes and red nodes represent AAA disease genes.
(EPS)

S3 Fig GO functional annotation of candidate disease genes. The red nodes are significantly overrepresented by candidate disease genes.
(EPS)

S1 Table Primer sequences used for Real-Time PCR. (XLS)

S2 Table List of candidate disease genes. (XLS)

S3 Table List of pathways enriched for level-2 candidate disease genes. (XLS)

## References

[1] Lindsay ME, Dietz HC. Lessons on the pathogenesis of aneurysm from heritable conditions. Nature. 2011;473(7347):308–16. 10.1038/nature10145 21593863 PMC3622871

[2] Zhang B, Kirov S, Snoddy J. WebGestalt: an integrated system for exploring gene sets in various biological contexts. Nucleic acids research. 2005;33(Web Server issue):W741–8. 15980575 10.1093/nar/gki475 PMC1160236

[3] Goh KI, Cusick ME, Valle D, Childs B, Vidal M, Barabasi AL. The human disease network. Proceedings of the National Academy of Sciences of the United States of America. 2007;104(21):8685–90. 17502601 10.1073/pnas.0701361104 PMC1885563

[4] Xu J, Li Y. Discovering disease-genes by topological features in human protein-protein interaction network. Bioinformatics. 2006;22(22):2800–5. 16954137 10.1093/bioinformatics/btl467

[5] George RA, Liu JY, Feng LL, Bryson-Richardson RJ, Fatkin D, Wouters MA. Analysis of protein sequence and interaction data for candidate disease gene prediction. Nucleic acids research. 2006;34(19):e130 17020920 10.1093/nar/gkl707 PMC1636487

[6] Hishigaki H, Nakai K, Ono T, Tanigami A, Takagi T. Assessment of prediction accuracy of protein function from protein—protein interaction data. Yeast. 2001;18(6):523–31. 11284008 10.1002/yea.706

[7] Titz B, Schlesner M, Uetz P. What do we learn from high-throughput protein interaction data? Expert review of proteomics. 2004;1(1):111–21. 15966804 10.1586/14789450.1.1.111

[8] Schwikowski B, Uetz P, Fields S. A network of protein-protein interactions in yeast. Nature biotechnology. 2000;18(12):1257–61. 11101803 10.1038/82360

[9] Bradford JR, Westhead DR. Improved prediction of protein-protein binding sites using a support vector machines approach. Bioinformatics. 2005;21(8):1487–94. 15613384 10.1093/bioinformatics/bti242

[10] Brown MP, Grundy WN, Lin D, Cristianini N, Sugnet CW, Furey TS, et al Knowledge-based analysis of microarray gene expression data by using support vector machines. Proceedings of the National Academy of Sciences of the United States of America. 2000;97(1):262–7. 10618406 10.1073/pnas.97.1.262 PMC26651

[11] Zhang L, Li X, Tai J, Li W, Chen L. Predicting candidate genes based on combined network topological features: a case study in coronary artery disease. PloS one. 2012;7(6):e39542 10.1371/journal.pone.0039542 22761820 PMC3382204

[12] Gao S, Wang X. Predicting Type 1 Diabetes Candidate Genes using Human Protein-Protein Interaction Networks. Journal of computer science and systems biology. 2009;2:133 20148193 10.4172/jcsb.1000025 PMC2818071

[13] Chua HN, Sung WK, Wong L. Exploiting indirect neighbours and topological weight to predict protein function from protein-protein interactions. Bioinformatics. 2006;22(13):1623–30. 16632496 10.1093/bioinformatics/btl145

[14] Samanta MP, Liang S. Predicting protein functions from redundancies in large-scale protein interaction networks. Proceedings of the National Academy of Sciences of the United States of America. 2003;100(22):12579–83. 14566057 10.1073/pnas.2132527100 PMC240660

[15] Chua HN, Ning K, Sung WK, Leong HW, Wong L. Using indirect protein-protein interactions for protein complex predication. Computational systems bioinformatics / Life Sciences Society Computational Systems Bioinformatics Conference. 2007;6:97–109. 17951816

[16] McKusick VA. Mendelian Inheritance in Man and its online version, OMIM. American journal of human genetics. 2007;80(4):588–604. 17357067 10.1086/514346 PMC1852721

[17] Becker KG, Barnes KC, Bright TJ, Wang SA. The genetic association database. Nature genetics. 2004;36(5):431–2. 15118671 10.1038/ng0504-431

[18] Prasad TS, Kandasamy K, Pandey A. Human Protein Reference Database and Human Proteinpedia as discovery tools for systems biology. Methods Mol Biol. 2009;577:67–79. 10.1007/978-1-60761-232-2_6 19718509

[19] Oti M, Brunner HG. The modular nature of genetic diseases. Clinical genetics. 2007;71(1):1–11. 17204041 10.1111/j.1399-0004.2006.00708.x

[20] Yamanouchi D, Morgan S, Stair C, Seedial S, Lengfeld J, Kent KC, et al Accelerated aneurysmal dilation associated with apoptosis and inflammation in a newly developed calcium phosphate rodent abdominal aortic aneurysm model. Journal of Vascular Surgery. 2012;56:455–61. 10.1016/j.jvs.2012.01.038 22560311 PMC3408782

[21] Shannon P, Markiel A, Ozier O, Baliga NS, Wang JT, Ramage D, et al Cytoscape: a software environment for integrated models of biomolecular interaction networks. Genome research. 2003;13(11):2498–504. 14597658 10.1101/gr.1239303 PMC403769

[22] Maere S, Heymans K, Kuiper M. BiNGO: a Cytoscape plugin to assess overrepresentation of gene ontology categories in biological networks. Bioinformatics. 2005;21(16):3448–9. 15972284 10.1093/bioinformatics/bti551

[23] Lenk GM, Tromp G, Weinsheimer S, Gatalica Z, Berguer R, Kuivaniemi H. Whole genome expression profiling reveals a significant role for immune function in human abdominal aortic aneurysms. BMC genomics. 2007;8:237 17634102 10.1186/1471-2164-8-237 PMC1934369

[24] Schubl S, Tsai S, Ryer EJ, Wang C, Hu J, Kent KC, et al Upregulation of protein kinase cdelta in vascular smooth muscle cells promotes inflammation in abdominal aortic aneurysm. The Journal of surgical research. 2009;153(2):181–7. 10.1016/j.jss.2008.04.032 18952226 PMC3322540

[25] Morgan S, Yamanouchi D, Harberg C, Wang Q, Keller M, Si Y, et al Elevated protein kinase C-delta contributes to aneurysm pathogenesis through stimulation of apoptosis and inflammatory signaling. Arteriosclerosis, thrombosis, and vascular biology. 2012;32(10):2493–502. 10.1161/ATVBAHA.112.255661 22879584 PMC3442600

[26] Wrana JL, Attisano L, Wieser R, Ventura F, Massague J. Mechanism of activation of the TGF-beta receptor. Nature. 1994;370(6488):341–7. 8047140 10.1038/370341a0

[27] Akool el S, Doller A, Babelova A, Tsalastra W, Moreth K, Schaefer L, et al Molecular mechanisms of TGF beta receptor-triggered signaling cascades rapidly induced by the calcineurin inhibitors cyclosporin A and FK506. J Immunol. 2008;181(4):2831–45. 18684975 10.4049/jimmunol.181.4.2831

[28] Nakao A, Afrakhte M, Moren A, Nakayama T, Christian JL, Heuchel R, et al Identification of Smad7, a TGFbeta-inducible antagonist of TGF-beta signalling. Nature. 1997;389(6651):631–5. 9335507 10.1038/39369

[29] Itoh S, Itoh F, Goumans MJ, Ten Dijke P. Signaling of transforming growth factor-beta family members through Smad proteins. European journal of biochemistry / FEBS. 2000;267(24):6954–67. 11106403 10.1046/j.1432-1327.2000.01828.x

[30] Daugherty A, Cassis LA. Mouse models of abdominal aortic aneurysms. Arteriosclerosis, thrombosis, and vascular biology. 2004;24(3):429–34. 14739119 10.1161/01.ATV.0000118013.72016.ea

[31] Golledge J, Muller J, Daugherty A, Norman P. Abdominal aortic aneurysm: pathogenesis and implications for management. Arteriosclerosis Thrombosis and Vascular Biology. 2006;26(12):2605–13. 10.1161/01.ATV.0000245819.32762.cb 16973970

[32] Golledge J, Kuivaniemi H, et al Genetics of abdominal aortic aneurysm. Current Opinion in Cardiology. 2013;28(3):290–6. 10.1097/HCO.0b013e32835f0d55 23478885

[33] Ortutay C, Vihinen M. Identification of candidate disease genes by integrating Gene Ontologies and protein-interaction networks: case study of primary immunodeficiencies. Nucleic acids research. 2009;37(2):622–8. 10.1093/nar/gkn982 19073697 PMC2632920

[34] Gunsalus KC, Ge H, Schetter AJ, Goldberg DS, Han JD, Hao T, et al Predictive models of molecular machines involved in Caenorhabditis elegans early embryogenesis. Nature. 2005;436(7052):861–5. 16094371 10.1038/nature03876

[35] Gandhi TK, Zhong J, Mathivanan S, Karthick L, Chandrika KN, Mohan SS, et al Analysis of the human protein interactome and comparison with yeast, worm and fly interaction datasets. Nature genetics. 2006;38(3):285–93. 16501559 10.1038/ng1747

[36] Jonsson PF, Bates PA. Global topological features of cancer proteins in the human interactome. Bioinformatics. 2006;22(18):2291–7. 16844706 10.1093/bioinformatics/btl390 PMC1865486

[37] Peters DG, Kassam AB, Feingold E, Heidrich-O’Hare E, Yonas H, Ferrell RE, et al Molecular Anatomy of an Intracranial Aneurysm Coordinated Expression of Genes Involved in Wound Healing and Tissue Remodeling. Stroke. 2001;32:1036–42. 11283408 10.1161/01.str.32.4.1036

[38] Li B, Li F, Chi L, Zhang L, Zhu S. The Expression of SPARC in Human Intracranial Aneurysms and Its Relationship with MMP-2/-9. PLOS ONE. 2013;8(3):e58490 10.1371/journal.pone.0058490 23516489 PMC3597740

[39] Kassam AB, Horowitz M, Chang YF, Peters D. Altered Arterial Homeostasis and Cerebral Aneurysms: A Molecular Epidemiology Study. Neurosurgery. 2004;54(6):1450–62. 15157303 10.1227/01.neu.0000125005.67850.f8

[40] Michineau S, Franck G, Wagner-Ballon O, Dai J, Allaire E, Gervais M. Chemokine (C-X-C Motif) Receptor 4 Blockade by AMD3100 Inhibits Experimental Abdominal Aortic Aneurysm Expansion Through Anti-Inflammatory Effects. Arteriosclerosis Thrombosis and Vascular Biology. 2014;37:1747–55. 10.1161/ATVBAHA.114.303913 24876351

[41] Abdul-Hussien H, Soekhoe RGV, Weber E, von der Thusen JH, Kleemann R, Mulder A, et al Collagen Degradation in the Abdominal Aneurysm: A Conspiracy of Matrix Metalloproteinase and Cysteine Collagenases. The American Journal of Pathology. 2007;170(3):809–17. 17322367 10.2353/ajpath.2007.060522 PMC1864891

[42] Obama T, Tsuji T, Kobayashi T, Fukuda Y, Takayanagi T, Taro Y, et al Epidermal growth factor receptor inhibitor protects against abdominal aortic aneurysm in a mouse model. Clinical science. 2015;128(9):559–65. 10.1042/CS20140696 25531554

[43] Leeper NJ, Raiesdana A, Kojima Y, Chun HJ, Azuma J, Maegdefessel L, et al MicroRNA-26a is a novel regulator of vascular smooth muscle cell function. Journal of cellular physiology. 2011;226(4):1035–43. 10.1002/jcp.22422 20857419 PMC3108574

[44] Golledge J, Biros E, Warrington N, Jones GT, Cooper M, van Rij AM, et al A population-based study of polymorphisms in genes related to sex hormones and abdominal aortic aneurysm. European journal of human genetics: EJHG. 2011;19(3):363–6. 10.1038/ejhg.2010.182 21119710 PMC3062004

[45] Liao M, Xu J, Clair AJ, Ehrman B, Graham LM, Eagleton MJ. Local and systemic alterations in signal transducers and activators of transcription (STAT) associated with human abdominal aortic aneurysms. The Journal of surgical research. 2012;176(1):321–8. 10.1016/j.jss.2011.05.041 21764069 PMC3197955

